# Perirenal Adipose Tissue Displays an Age-Dependent Inflammatory Signature Associated With Early Graft Dysfunction of Marginal Kidney Transplants

**DOI:** 10.3389/fimmu.2020.00445

**Published:** 2020-03-17

**Authors:** Romain Boissier, Pauline François, Bastien Gondran Tellier, Maité Meunier, Luc Lyonnet, Stephanie Simoncini, Jeremy Magalon, Tristan Legris, Laurent Arnaud, Laurent Giraudo, Françoise Dignat George, Gilles Karsenty, Stéphane Burtey, Eric Lechevallier, Florence Sabatier, Pascale Paul

**Affiliations:** ^1^Department of Urology and Renal Transplantation, La Conception University Hospital, Assistance Publique-Hôpitaux Marseille (APHM), Aix-Marseille Univ., Marseille, France; ^2^C2VN, INSERM 1263, Aix-Marseille Univ, INRAE, Marseille, France; ^3^Cell Therapy Department, La Conception University Hospital APHM, Aix-Marseille Univ., INSERM CIC 1409, Marseille, France; ^4^Department of Nephrology and Renal Transplantation, La Conception University Hospital, Assistance Publique-Hôpitaux Marseille (APHM), Aix-Marseille Univ., Marseille, France; ^5^Department of Hematology and Vascular biology, La Conception University Hospital, Assistance Publique-Hôpitaux Marseille (APHM), Aix Marseille Univ., Marseille, France

**Keywords:** marginal kidney donors, kidney transplantation, natural killer cells, endothelial inflammation, perirenal adipose tissue, kidney allograft dysfunction

## Abstract

**Background:** Better understanding of the contribution of donor aging and comorbidity factors of expanded criteria donors (ECD) to the clinical outcome of a transplant is a challenge in kidney transplantation. We investigated whether the features of donor-derived stromal vascular fraction of perirenal adipose tissue (PRAT-SVF) could be indicative of the deleterious impact of the ECD microenvironment on a renal transplant.

**Methods:** A comparative analysis of cellular components, transcriptomic and vasculogenic profiles was performed in PRAT-SVF obtained from 22 optimal donors and 31 ECD deceased donors. We then investigated whether these parameters could be associated with donor aging and early allograft dysfunction.

**Results:** When compared with the PRAT-SVF of non-ECD donors, ECD PRAT-SVF displayed a lower proportion of stromal cells, a higher proportion of inflammatory NK cells. The global RNA sequencing approach indicated a differential molecular signature in the PRAT-SVF of ECD donors characterized by the over-expression of CXCL1 and IL1-β inflammatory transcripts. The vasculogenic activity of PRAT-SVF was highly variable but was not significantly affected in marginal donors. Periorgan recruitment of monocytes/macrophages and NK cells in PRAT-SVF was associated with donor aging. The presence of NK cell infiltrates was associated with lower PRAT-SVF angiogenic activity and with early allograft dysfunction evaluated on day 7 and at 1 month post-transplant.

**Conclusions:** Our results indicate that human NK cell subsets are differentially recruited in the periorgan environment of aging kidney transplants. We provide novel evidence that PRAT-SVF represents a non-invasive and timely source of donor material with potential value to assess inflammatory features that impact organ quality and function.

## Introduction

Increasing recipient demand combined with inadequate organ supply has led to the use of suboptimal marginal kidneys from expanded criteria donors (ECD) with cardiovascular risk factors (1–3). While the use of ECD kidney transplants enables more patients to benefit from renal transplantation, various studies have reported that marginal transplants from elderly donors are associated with an increased incidence of delayed graft function (DGF), slow graft function recovery (SGF) (3–6) and poorer long-term graft outcome (5, 7–9).

The underlying mechanisms which associate donor age and cardiovascular risk factors with a worsened outcome of these marginal transplants are not completely understood. Since graft endothelial cells constitute the critical interface between the donor and the recipient, pre-existing endothelial dysfunction of the donor could be considered as an initial checkpoint leading to deleterious recipient immune responses and vascular rejection (10–13). Several studies have highlighted the “higher immunological risk” of transplants from marginal donors resulting in stress-induced senescence mechanisms (14, 15) and induction of endothelial adhesion and inflammatory molecules (16–19).

The current challenge is therefore to delineate the donor-related features that determine the capacity of transplant endothelium to resist further exposure to ischemia, oxidative, uremic and alloimmune inflammatory stresses associated with the transplant procedure. There is a lack of models that identify donor-related features that reflect the endothelial quality of aging ECD transplants (16).

Our study is based on the hypothesis that perirenal adipose tissue (PRAT), systematically discarded during the surgical preparation of a renal transplant, represents an easily accessible source of donor-derived material allowing assessment of the quantitative and functional features that characterize exposure of donor cells to the ECD microenvironment.

Indeed, adipose tissue (AT) can be enzymatically processed to yield stromal vascular fraction (SVF), a heterogeneous cellular mixture devoid of adipocytes that recapitulates the variety of cells that constitute the vasculature such as mesenchymal stem cells, pericytes, endothelial progenitor cells and leucocytes. Endothelial progenitor cells, also called endothelial colony forming cells (ECFC), have been recently identified in the vessel wall and SVF (20–22). We and others have reported that the phenotypic and angiogenic activity of ECFC can be altered in deceased contexts associated with cardiovascular risk factors or genetic or epigenetic determinants (23–25). Furthermore, it was recently demonstrated that donor age and comorbidities can alter the angiogenic and paracrine immunosuppressive properties of human bone marrow-derived stromal cells (BM-SC) obtained after *in vitro* cell culture expansion (26). It is thus likely that the ECD microenvironment can also alter the vascular potential of the various types of PRAT SVF-resident cells.

Based on this knowledge, we postulated that donor PRAT-SVF could represent a relevant and non-invasive model to evaluate the ECD microenvironment factors that could contribute to the alteration of renal transplant quality. This study aimed to (1) provide a comprehensive view of cellular, transcriptomic, and angiogenic profiles that could characterize the peri-organ SVF obtained from marginal kidney donors, and (2) analyze whether the features of PRAT-SVF could be indicative of the deleterious impact of donor aging and cardiovascular risk factors on early kidney allograft dysfunction.

## Materials and Methods

### Patients and Sample Collection

We conducted a monocentric prospective study involving 53 renal transplantation procedures performed in the Department of Urology and Renal transplantation, La Conception University Hospital in Marseille, France from 2016 to 2018. For each renal transplant, the stromal vascular fraction (PRAT-SVF) was isolated from the perirenal AT collected during kidney procurement and submitted to analysis of cellular components, transcriptomic profile and vasculogenic activity. The study was approved by the National Ethics Committee of the Agence de la Biomédecine (ABM), the National Ministry of Research and adhered to the Jardé Law on human investigation. All procedures were conducted in compliance with the Declarations of Helsinki and Istanbul. Data were prospectively and anonymously collected in a dedicated database for the exclusive access of the authorized authors.

### Clinical Variables

The following demographic data were recorded for donors and recipients: sex, age, body mass index, blood group, serum creatinine, cardiovascular risk factors (history of smoking, hypertension, dyslipidemia, diabetes mellitus, coronary heart disease. Renal function (serum creatinine, glomerular filtration rate) were recorded at D7, M1, and M12 during renal transplantation follow-up. The CKD EPI formula was used to evaluate renal function in adults and the Schwartz formula was used in younger recipients (<18 years) (27).

### Definition of Endpoints

ECD kidney transplants were defined as those from donors aged ≥60 years or 50 to 59 years with 2 of the following comorbidities: hypertension, serum creatinine >1.5 mg/dl, or death following cerebrovascular accident.

Delayed graft function (DGF) was defined as the use of dialysis within 7 days of the transplant (28). Slow graft function (SGF) was defined by serum creatinine > 250 umol/L (3.0 mg/dL) on postoperative day 7 (29).

### Identification of Anti-HLA Antibodies

The detection of HLA-specific antibodies was performed using standard techniques. The presence of allograft- specific antibodies was screened through Luminex screening assays (LAScreen® mixed, One Lambda, Canoga Park, CA, USA) using Luminex flow beads (LAScan™ 100, Luminex, Austin, TX, USA). To determine their antibody specificity, all samples with a positive screening result were further evaluated using single-antigen flow bead assays according to the manufacturer's recommended protocol (LAScreen® Single Antigen class I or LAScreen® Single Antigen class II, One Lambda, Canoga Park, CA, USA). The percentage of HLA sensitization for the single-antigen assays were calculated according to the manufacturer's instructions as the percentage of positive bead reactions among the 99 class I beads and 97 class II beads.

### Isolation of the Stromal Vascular Fraction From Donor Perirenal AT

Perirenal adipose tissue (at least 30 g) was collected under aseptic conditions during the multi-organ retrieval for cadaveric donor renal transplantation. Excised fat was manually sliced with scissors into units of ~3 × 3 × 3 mm. Enzymatic digestion was performed using 0.25UI/mL collagenase NB4 (Serva, Heidelberg, Germany) for 1 h at 37°C under constant agitation. Three cycles of wash/centrifugation were performed to eliminate adipose and red blood cells. Freshly isolated PRAT-SVF was used for flow cytometry analysis and RNA extraction and cryopreserved at −180°c for delayed angiogenic assays performed on thawed samples.

### Phenotypic Characterization of PRAT- SVF Cell Subsets

Multiparameter flow cytometry analysis was performed to compare the quantitative distribution of the CD45- and CD45+ cell subsets in the donor-derived PRAT-SVF in the non ECD and ECD groups.

Half a million cells per tube were suspended in 100 μL of phosphate-buffered saline (PBS, Gibco®, Life Technologies), stained 20 min at room temperature and protected from light with the DRAQ5 nuclear marker, the NucBlue viability marker and two pre-prepared antibody mixes or corresponding isotype controls in matched concentrations. The first monoclonal antibody mix contained the following surface markers: CD146, CD34, and CD45, labeled respectively with the following fluorochromes: PE, ECD, and PC5. The second mix was composed of the following surface markers: CD14, CD34, CD45, CD56, CD3 labeled respectively with FITC, ECD, PC5, PC7 and APC-Alexa Fluor 750 fluorochromes (References in Supplementary Table 1). Flow cytometry was performed with a NAVIOS instrument (Beckman Coulter, Brea, California, USA). Data files were analyzed using Kaluza software (Beckman Coulter, Brea, California, USA). The gating strategy used to identify the various cell subsets is summarized in Supplementary Figure 1.

### RNA Purification and RNAseq Gene Expression Analysis

Total RNA was isolated from PRAT-SVF using RNeasy mini kits (QIAGEN Inc., Valencia, CA, USA) including a DNase I digestion step removing genomic DNA. RNAseq analysis of SVF profiles in ECD vs. non-ECD kidney donors was performed by HalioDX. Briefly, the purity and concentration of the samples were estimated by spectrophotometer. The integrity of the RNA (RIN > 8) was evaluated on an RNA 6000 Nano LabChiprun Agilent 2100 Bioanalyzer (Agilent technologies, Germany). Generation of libraries was performed using PerklinElmer technologies with the NEXTflex qRNA-Seq kit v2 after total RNA enrichment by NEXTflex Poly(A) beads following manufacturer's recommendations (PerkinElmer). RNA-seq library were sequenced on Illumina Nextseq sequencer. The generated reads were single-end and of 76-nt length. FastQC (version 0.11.5) was used to examine the read quality. Trimming of reads was performed using Trimmomatic (version 0.33) on the base of an average phred quality of 20. The raw single-ends reads were then mapped against the human genome (GRCh38.90) from the Ensembl database using STAR (version 2.5.3a) sequence mapper. The resulting BAM files were examined by Qualimap (version 2.2). Duplicates reads were removed using the function MarkDuplicates of picard tools (version 2.9.0). Unduplicated reads were used to count reads per gene with FfeatureCounts (version 1.5.2). Raw counts are converted in reads per Million (RPKM) and log transformed (log base 2) in order to help with distributional assumptions, linearity and consistency with PCR based methods for calculating the Fold Change. Genes of interest were filtered using a mean RPKM >25 and a coefficient of variation >50% (1183 genes passed out).

R/Bioconductor packages including DESeq2 were used for gene expression analysis. Finally, we selected differentially expressed genes with a *P*-value < 0.05 and a Fold Change (FC) of a least 1.5.

Genes up or downregulated were separately subjected to functional annotation analysis using the Database for Annotation Visualization and Integrated Discovery (DAVID, david.ncifcrf.gov/) online tool to find significantly enriched genes biological functions and associated pathways. Gene Ontology: Biological Process and KEGG (Kyoto Encyclopedia of Genes and Genomes) pathway enrichment analysis was performed with a cut-off criteria for the threshold of EASE score <0.05 (modified Fisher Exact *P*-value).

### Real Time PCR Analysis of Transcripts

Total RNA (5 μg) was converted to cDNA using 200U of M-MLV reverse transcriptase (Invitrogen). Real-time PCR amplification was performed with the Light Cycler 480 SYBR Green I Master kit (Roche). Cycling conditions were 10 min at 95°C (hot-start PCR), followed by 40 cycles, 10 s at 95°C (denaturation), 15 s at 62°C (annealing) and 20 s at 72°C (elongation). Melting curve analysis was performed to check the specificity of amplification. Reported values are relative numbers of specific transcripts detected per 10^6^ *GAPDH* transcripts. The primers used for gene-specific amplification are described in Supplementary Table 2.

### Tube Formation Assay

Tube formation assay was analyzed *in vitro* using PRAT-SVF in Matrigel^TM^ (6 mg/ml) (Corning® Matrigel® Basement Membrane Matrix Growth Factor Reduced, Phenol Red Free, 356231) as described by Zakhari et al. (30). The PRAT-SVF from ECD and non-ECD donors were loaded at a density of 20,000 cells/well in a μ-slide angiogenesis (81506, IBIDI) system coated with 10 μl of growth factor-reduced Matrigel^TM^ (6 mg/ml) (Corning® Matrigel® Basement Membrane Matrix Growth Factor Reduced, Phenol Red Free, 356231), previously polymerized for 30 min, and were maintained in endothelial basal cell culture medium-2 (EBM2) supplemented with MV SingleQuots (EGM2-MV) (Lonza, Clonetics, Walkersville, MD, USA) at 37°C with 5% CO_2_. Capillary-like structures were recorded after 72 h using a Leica DMI8 video-imaging inverted microscope equipped with an Incubator I8 at 5X magnification and were captured using Leica Application Suite X software (Las X 3.0.2.16120). As previously described (30), various parameters that reflect the relevant steps of SVF vasculogenic/angiogenic capacity *in vitro*, were quantified: the number of clusters, indicative of the capacity of the plated cells to self-assemble; the number of clusters with tip cells, indicative of the ability of cells to undergo specialization into cells able to migrate away from the cluster and initiate sprouting; the number of clusters with stalk cells that represent the capacity of cells to proliferate and elongate neovessels; and the number of branching points that provide information on the capacity of cells to develop as complex vascular networks. Cell clusters were automatically counted using Fiji software under cellular analysis with minimum object size set at 500 μm and a maximum object size set at 3,000 μm. These counts along with manual counts of tip cells, stalk cells and branch points were taken using still frames of both groups. Each experiment was performed in triplicate.

### Spheroid-Based Sprouting Assay

Angiogenic sprouting was analyzed *in vitro* using PRAT-SVF in a collagen gel matrix as previously described by Korff et al. (31). The images were then analyzed using the Sprout Analysis plug-in developed by Eglinger et al. (32) in the Fiji distribution of ImageJ, to evaluate the different vascular parameters, such as sprout length and branch points.

PRAT-SVF from ECD, non-ECD deceased donors and living donors were suspended in culture medium containing 0.2% (wt/vol) carboxymethylcellulose (M0512, Sigma, Munich, Germany), which was then seeded in non-adherent round-bottom 96-well plates (82.1582.001, Sartstedt), leading to the formation of spheroids with a defined cell number. After 72 h, the spheroids were collected and embedded in collagen gels (354236, Corning® Collagen I, Rat Tail). The spheroid containing gel was rapidly transferred into pre-warmed Labtek II slides (NUNC 54534, ThermoFisher) and allowed to polymerize (30 min), then 100 μl of EGM2-MV medium were added on the top of the gel. Following 24 h of culture in EGM2-MV medium, the spheroids were fixed for 30 min in 4% paraformaldehyde at room temperature. After washing and permeabilization 2 h at 4°C with PBS containing 0.1% Triton X-100 and 1% BSA, the spheroids were immunolabeled overnight at 4°C with phalloidin coupled with Alexa-647 (A22287, ThermoFisher Scientific) (1/100), and nuclei were stained with 6-diamidino-2-phenylindole (DAPI) (1/5000) diluted in PBS 1% BSA. After washing, we then captured a fluorescent optical image stack along the z-axis at 20X magnification using two lasers in sequential mode under a Leica DMI8 microscope (at least n = 10 spheroids per condition). Las X software was used during all image acquisition procedures. Image processing prior to image measurements was performed with Huygens Essential deconvolution software (Scientific Volume Imaging,) using up to 40 iterations of the classical maximum likelihood estimation algorithm, with a theoretical PSF and automatic background correction. The images were then analyzed using the Sprout Analysis plug-in developed by Eglinger et al. (32) in the Fiji distribution of ImageJ, to evaluate the different vascular parameters, such as sprout length and branch points.

### Statistical Analysis

Statistical analysis comparing continuous variables in 2 groups was performed using non-parametric Wilcoxon–Mann–Whitney test and categorical variables with Chi2 test using Xlstat® version 2018.5 (Addinsoft, Paris, France) and Graphpad Prism® version 7 (GraphPad Software, California, USA). Categorical variables were presented as frequencies and continuous variables as the mean ± standard deviation (SD) or median and 10–90 or 25–75 percentile according to the test of normal distribution using the Kolmogorov-Smirnov test. Spearman rank correlation was used to evaluate the associations between quantitative parameters analyzed in PRAT-SVF and age or creatinine or CKD evaluated in the recipient at D7 and M1 post-transplant. Only variables with *P* < 0.20 were considered. Significant differences were considered when the *P*-value was < 0.05. Univariate and multivariate logistic regression analyses were performed to evaluate whether the PRAT-SVF parameters evaluated could discriminate the effect of aging (<59 years) or transplants that had good functional recovery from those with impaired graft function (based on the use of the 60 and 45 ml/ min per 1.73m^2^ CKD eGFR cut-off values corresponding to moderate or mild CKD at M1). The AUC of the receiver operating characteristic (ROC) curve was used to define the threshold of quantitative variables that best predicted early dysfunction of the transplant at M1 post-transplant.

## Results

### Donor and Recipient Characteristics

Fifty-three donors were included: 31 (49%) expanded criteria donors (ECD) and 22 (35%) non-expanded criteria donors (non-ECD). Donor characteristics are summarized in Table 1. Compared with non-ECD, the ECD group presented with higher age (71 vs. 42 years, *P* < 0.01) and a higher prevalence of cardiovascular risk factors (hypertension, dyslipidemia, vasculopathy, all *P* < 0.05) except for the prevalence of diabetes that did not reach significance Second transplant concerned 7% of the analyzed cohort. All the patients received the same induction therapy except for one patient in the non ECD donor group that was treated with anti IL2. Rabbit antithymocyte globulin (rATG) were administered on day 0 (1.25 g/kg/day) for 8 days. Prednisolone was administered on day 0 (initially 1 mg/kg/day), with subsequent tapering to achieve a targeted mean maintenance dose of 0.25 mg/kg/day at day 30 after transplant. As previously described (33), maintenance immunosuppressive therapy associated Tacrolimus/mycophenolate mofetil (FK/MMF, 64%) or ciclosporine/azathioprine (CSA/Aza, 36%) immunosuppressive combination. All recipients transplanted with an optimal kidney (non ECD) were treated with FK/MMF maintenance therapy, while the CSA/Aza immunosuppressive combination was used in 62 % of the recipients transplanted with an ECD kidney (Table 1).

**Table 1:** Donors and recipients characteristics.

	**ECD**	**Non-ECD**	***P*-value**
	***n* = 31**	***n* = 22**	
**DONOR BASELINE CHARACTERISTICS**			
Gender (M/F) %	55/45 %	59/41%	0.79
Age, years (median, 25–75 IR)	71 [65–78]	42 [30–53]	<0.01
BMI, kg/m^2^ (median, 25–75 IR)	27 [24–29]	24 [22–27]	0.03
**DONORS RENAL FUNCTION**			
Serum creatinine (micromoles/L)	78 [60–98]	67 [48–100]	0.39
Proteinuria, g/L	0.2 [0.1–0.5]	0.2 [0.1–0.5]	0.65
**DONOR MEDICAL HISTORY**			
Smoking %	7%	50%	<0.01
Hypertension %	61%	0%	<0.01
Dyslipidemia %	26%	0%	0.02
Diabetes mellitus %	10%	5%	0.63
Vasculopathy %	29%	0%	0.01
Cold ischemia time, hours	12 [9–15]	13 [10–17]	0.33
Side (Left/Right), %	89/11%	80/20%	0.63
**RECIPIENT BASELINE CHARACTERISTICS**			
Gender (M/F) %	58/42%	82/18%	0.57
Age, years (median, 25–75 IR)	67 [61–73]	39 [28–52]	<0.01
BMI, kg/m^2^ (median, 25–75 IR)	25 [22–27]	23 [19–26]	0.07
**RECIPIENT MEDICAL HISTORY**			
Hemodialysis %	84%	85%	0.99
Dialysis duration, months	35 [17–47]	32 [2–53]	0.64
Smoking %	33%	10%	0.08
Hypertension %	92%	70%	0.11
Dyslipidemia %	25%	20%	0.73
Diabetes mellitus %	25%	5%	0.11
Coronary heart disease %	29%	10%	0.15
**PRETRANSPLANT ASSESSMENT**			
HLA class I and/or class II sensitization (%)	35%	39%	0.79
% of HLA class I positive beads	5% [5–7]	5% [5–5]	0.99
% of HLA class II positive beads	26% [13–39]	6% [6–6]	0.26
Rank of renal transplantation >1	9%	5%	0.63
**MAINTENANCE IMMUNOSUPRESSIVE TREATMENT**			
Steroids/Tacrolimus/Mycofenolate Mofetil	38.5%	100%	<0.01
Steroids/Ciclosporin/Azathioprine	61.5%	0%	
**RENAL GRAFT OUTCOME**			
Slow/delayed graft function %	45%	14%	0.02
**Graft function day 7 (D7)**			
Serum creatinine (micromoles/L)	374 [146–573]	171 [66–178]	<0.01
eGFR (mL/min/1.73m^2^)	25 [6–40]	67 [38–111]	<0.01
**Graft function at month 1 (M1)**			
Serum creatinine (micromoles/L)	162 [111–193]	103 [72–129]	<0.01
eGFR (mL/min/1.73m^2^)	41 [23–50]	82 [68–120]	<0.01
Mean time Follow-up, months	13.5	11.6	0.62

*Values are reported as % or median [25–75 interquartile ranges]*.

The recipients of ECD kidney grafts were significantly older than the non-ECD recipients (median 67 vs. 39 years, *P* < 0.01). However, the prevalence and duration of dialysis before kidney transplantation were comparable in both groups. While most patients were negative for anti HLA panel reactive antibodies (PRA) evaluated before graft (63 %), HLA class I PRA sensitization was observed in 29% of the patients, anti HLA class II sensitization without Class I immunization in 2% of the patients and combined immunization against class I and Class II in 5% of kidney transplant recipients. None of the anti HLA antibodies detected before transplant were Donor specific antibodies (DSA) and transplant recipients analyzed in the study cohort did not develop *de novo* DSA during the first 3 months following transplant. The incidence of slow/delayed graft function (SDGF) was significantly higher in the recipients of ECD allografts (45% vs. 14%, *P* = 0.02). The average glomerular filtration rate (eGFR) of ECD donor kidney grafts was significantly lower when compared to non-ECD kidney grafts on day 7 (D7) and at 1 month (M1) post-transplant (with eGFR of 25 vs. 67 ml/min/1.73m^2^ on D7 and 41 vs. 82 mL/min/1.73m^2^ at M1, all *P* < 0.05). Mean follow-up post-transplantation period was 12.7 months.

### The Cell Subset Distribution of Perirenal SVF Is Altered in ECD Donors

**Figure 1:**
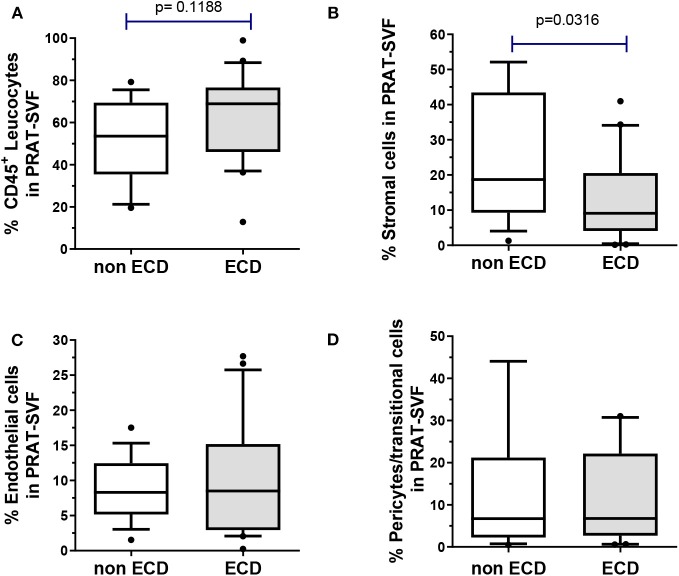
The distribution of cell subsets composing the PRAT-SVF was determined using flow cytometry and compared between the non-ECD and ECD donors: **(A)** CD45^+^ leukocytes were comparable in the two groups **(B)** mesenchymal stem/stromal cells were significantly lowered in the ECD vs. non-ECD group **(C)** pericytes and transitional cells **(D)** and endothelial cells were not statistically different between the two groups. ECD, extended criteria donors. Non ECD, non-extended criteria donors. Results on the graphs are reported as box and whiskers plots representative of median values, and 25–75 interquartile ranges (Boxes) and error bars indicative of 10-90 percentile ranges. Dots indicates values out of the 10–90% quartile range.

High inter-individual variability in the distribution of SVF cell subsets was observed among the donors (Figure 1). The CD45+ leucocyte population was the most prevalent subset (median 61 %, 25–75 percentile range 47–74) and tended to be higher in the ECD donors (Figure 1A). The median percentage of stromal cells (13%, 25–75 percentile: 7–30%) was significantly lower in the ECD donors (9 %) when compared with the non-ECD donors (18%, *p* = 0.03) (Figure 1B). The quantitative distribution of endothelial cells (median 8.5 %, 25–75 percentile: 4–13%, Figure 1C) and pericytes (median 7%, 25–75 percentile: 2.4–22.1, Figure 1D) were comparable in PRAT-SVF from the two groups of kidney donors.

### Comparative Analysis of PRAT-SVF the Angiogenic Activity of ECD and Non-ECD Donors

**Figure 2:**
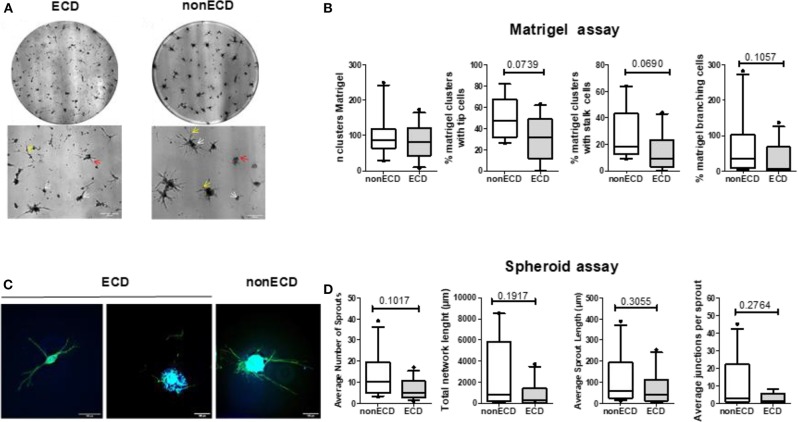
**(A)** Representative experiment of capillary tube formation by SVF from ECD or non-ECD. A total of 20,000 cells/well were seeded on growth factor reduced Matrigel. Images were recorded at 72 h with a phase-contrast microscope. Original magnification x5; upper panel (scale bars, 300 μm) correspond to the total image of the well while the lower panels were zooms of previous images (scale bars, 200 μm). White arrows identify initial cell clustering; red arrows marked the tip cells while yellow arrows identified branching. **(B)** Quantitative analysis of number of clusters, percentage of clusters with tip and stalk cells, number of branching points. Data are expressed as means ± SEM of independent experiments performed in triplicate using PRAT-SVF obtained in 14 ECD and 10 non-ECD donors **(C)** Representative experiment of 3D *in vitro* angiogenic assay with collagen gel-embedded spheroids of SVF from ECD or non-ECD (original magnification x20; scale bars, 100 μm). Imaging of Vascular sprouts was obtained after merging of actin staining (phalloidin in green) and nuclei staining (DAPI, blue) as detailed in Supplementary Figure 2 **(D)** Quantitative analysis of number of sprouts, branch points, and total network length per spheroid as well as average sprout length was compared in PRAT-SVF from the ECD and non ECD donors. For each experiment, at least 10 spheroids were analyzed.

The SVF-dependent formation of capillary-like structures, evaluated in an *in vitro* Matrigel^TM^ assay (Figure 2A) as the number of clusters, was similar in the ECD and non-ECD donors (Figure 2B). A trend toward a decrease in the tip cell (*P* = 0.07) as well as stalk cell (*P* = 0.07) percentages was observed for ECD PRAT-SVF, but did not reach significance (Figure 2B). In addition, the vessel complexity characterized by the Matrigel branching points was also preserved in the ECD donor PRAT-SVF (Figure 2B). In a 3D spheroid assay (Figure 2C), the sprout formation, total network length and the average number of junctions formed by sprouts presented a trend toward a decrease in ECD PRAT-SVF, when analyzed in reference to the non-ECD donor PRAT-SVF (Figure 2D and Supplementary Figure 2).

Taken together, these data suggest a high inter-individual heterogeneity in the angiogenic potential of donor PRAT-SVF but does not identify a significant impairment of the median angiogenic activity of PRAT-SVF derived from ECD donors, when compared to ECD donors.

### Transcriptomic Analysis of PRAT-SVF Identified Inflammatory Profiles Specific to ECD Donors

**Figure 3:**
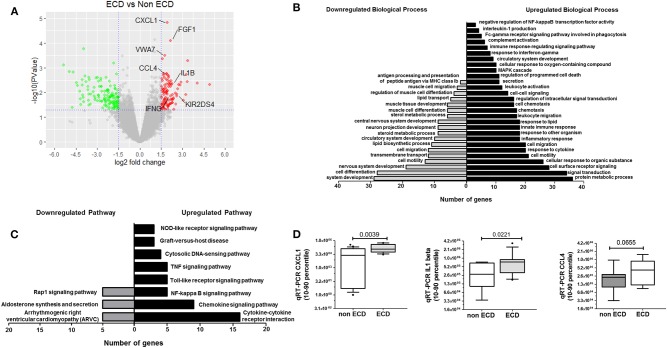
Comparative Transcriptomic analysis of SVF from ECD (*n* = 5) and non-ECD (*n* = 5) patients. **(A)** Volcano plot of differentially expressed genes from SVF from ECD and non-ECD. Log2 Fold Change value obtained by RNAseq plotted against the –log10 of *P*-value. Genes with a fold change > |1.5| and *P* < 0.05 were deemed to be differentially expressed. *P* = 0.05 is indicated by horizontal lines. Positive and negative fold change values are reflective of down-regulated (green) or up-regulated (red) genes compared with non-ECD condition, respectively. **(B)** Fold enrichment over chance for the Gene Ontology Biological process of the Down (gray) and Up (black) gene lists using DAVID (fold change > |1.5| and *P* < 0.01). **(C)** Fold enrichment over chance for the KEGG Pathway of the Down (gray) and Up (black) gene lists using DAVID (fold change > |1.5| and *P* < 0.01). **(D)** qRT-PCR validation of selected genes expressed a relative levels of specific transcripts detected per 10^6^ GAPDH transcripts [median (25–75% quartile)].

A comparative RNAseq transcriptomic analysis was performed to compare the PRAT-SVF molecular transcripts in ECD and non-ECD donors (Supplementary Table 3). Volcano plot distinguished a significant differential gene expression profile based on the comparison of ECD and non-ECD patients (Figure 3A). Overall, differential expression analysis revealed 245 genes showing fold change (FC) values ≥ 1.5 (111 genes overexpressed in ECD vs. non-ECD, Supplementary Table 4) and FC ≤ −1.5 (134 genes under-expressed in ECD vs. non-ECD) with *P* ≤ 0.05 (Supplementary Table 5). To provide a cohesive view of the biological functions associated with the changes in the ECD-SVF gene expression profile, we conducted a gene ontology analysis using the DAVID database. The up-regulated genes showed a strong association with the inflammatory response and cytokine secretion as well as circulatory system development Supplementary Table 6, whereas the categories enriched among the down-regulated genes were associated with the regulation of metabolic processes and the regulation of the circulatory system development (Figure 3B and Supplementary Table 7). Moreover, KEGG pathway analysis revealed differential inflammatory pathways, as “chemokine pathway,” “NF-kappa B pathway” or “TNF signaling pathway,” as well as “Graft-versus-host disease” (Figure 3C and Supplementary Tables 8, 9).

Based on their previous involvement in graft rejection or angiogenesis and the FC in their differential expression between ECD and non-ECD patients, five genes were selected (*CXCL1, VWA7, CCL4, IL1-*β*, IFN-*γ) for further quantitative RT-PCR (qPCR) validation in the PRAT-SVF samples used to perform transcriptomic analysis (Supplementary Table 10). Analysis of an extended number of PRAT-SVF samples derived from 12 non-ECD and 13 ECD additional PRAT-SVF samples showed highly variable transcript expression among donors and confirmed the enhanced levels of CXCL1, IL1-β transcripts in ECD donor PRAT-SVF (Figure 3D). Relative transcript levels of CCL4 tended to be higher in ECD PRAT-SVF (*p* = 0.07, Figure 3D). Thus, these data identified an enrichment of genes involved in the control of inflammatory responses.

### Donor Aging Is Associated With Inflammatory Profile in PRAT-SVF

**Figure 4:**
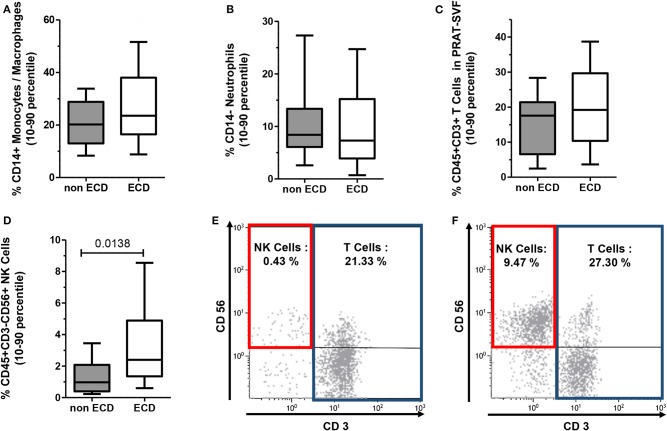
The distribution of CD45^+^ hematopoietic cells composing the PRAT-SVF of ECD and non-ECD donors was analyzed using flow cytometry according to the gating strategy illustrated in Supplementary Figure 1. Results on the graphs are reported as box and whiskers plots representative of median values, and 25–75 interquartile ranges (Boxes) and error bars indicative of 10–90 percentile ranges. **(A)** CD14+ Monocyte/Macrophage subset. **(B)** CD14-neutrophil subset. **(C)** CD3+ T lymphocytes. **(D)** CD3−CD56+ NK cells. **(E)** Histograms illustrate the gating and % of CD3+ T cells and CD3-CD56+ NK Cells observed when analyzing PRAT-SVF of a non-ECD donor (0.43% of NK Cells) or **(F)** PRAT-SVF from an ECD donor (9.47% of NK cells).

Transcriptomic data prompted further analysis of the distribution of inflammatory cells within CD45+ cell compartment of PRAT-SVF. While the distribution of CD45+CD14+ macrophages/monocytes (Figure 4A), CD45+CD14- neutrophils (Figure 4B), and CD45+CD3+ T lymphocyte subsets (Figure 4C) was comparable among the two groups, the percentage of CD45+CD3-CD56+ NK cells was significantly higher in ECD PRAT-SVF (median value: 2.8%, 25–75 percentile: 1.3–5.1%) compared to non ECD PRAT-SVF (0.97%, 0.4–2.1%, *p* = 0.01) (Figure 4D). Interestingly, the percentage of NK cells was further associated with the level of transcripts encoding INF-γ inflammatory cytokines and the activating NKG2D receptor (Table 2). Enhanced levels of NK cell infiltrates were also associated with parameters indicative of endothelial dysfunction such as lowered angiogenesis scores and FGFR2 transcript levels (Table 2).

**Table 2:** Analysis of the link between quantitative parameters evaluated in PRAT-SVF and % of PRAT-SVF NK cells.

**% CD3- CD56+ NK cell PRAT-SVF**	**Spearman r**	***P*-value**
**DONOR CHARACTERISTICS**		
Donor age	0.6228	0.0007^***^
**PRAT SVF IMMUNE CELLS**		
% CD3+ T cell PRAT-SVF	0.4845	0.0121^*^
% CD14+ Mono/macro PRAT-SVF	0.4216	0.0319^*^
**PRAT-SVF TRANSCRIPTS**		
PRAT-SVF NKG2D transcript Levels	0.676	0.0021^**^
PRAT-SVF FGFR2 transcript levels	−0.624	0.0098^**^
PRAT-SVF IFN-γ transcript levels	0.5611	0.0101^*^
**ANGIOGENESIS**		
Spheroid total network length	−0.5368	0.0178^*^
Spheroid average junction per sprout	−0.5035	0.028^*^
Spheroid number of sprouts	−0.4906	0.033^*^
Matrigel number of clusters	−0.4719	0.0413^*^

*BMI, Body Mass Index; PRAT, PeriRenal Adipose Tissue; SVF, Stromal Vascular Fraction*.

* *P < 0.05*,

** *P < 0.01*,

*** *P < 0.001*.

**Figure 5:**
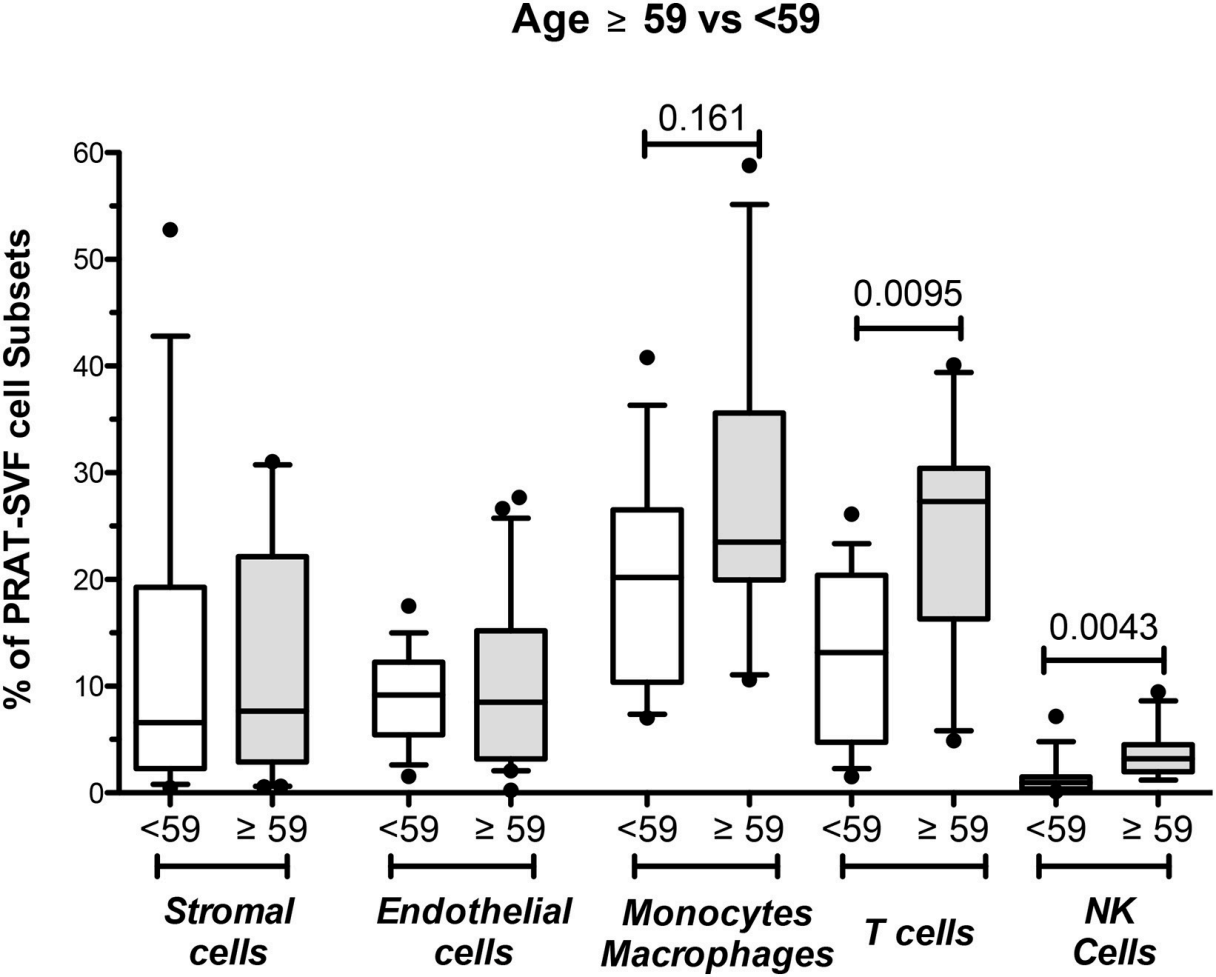
Analysis of perirenal adipose tissue stromal vascular fraction (PRAT-SVF) in young vs. aged donors. PRAT-SVF was analyzed according to donor age when stratified in aging donors (≥59 years, *n* = 29) and younger donors (<59 years, *n* = 24). Percentages in stromal and endothelial cells were not different between the aging and younger donors. However, the aging donors presented a trend for increased representation of the CD45+ CD14+ monocyte macrophage cell subset and a significantly higher percentage of T and NK cells.

Donor age was also statistically associated with an inflammatory profile characterized by a significantly higher percentage of NK cells in PRAT-SVF. Stratification of donors according to the 59-year median value observed in the cohort confirmed the increased percentage of NK and T cell lymphocytes in the PRAT-SVF of donors ≥59 years (Figure 5). Donor-related factors other than age could not be significantly associated with PRAT-SVF inflammatory profile.

### The NK Inflammatory Profile of PRAT SVF Is Associated With Early Allograft Dysfunction

**Figure 6:**
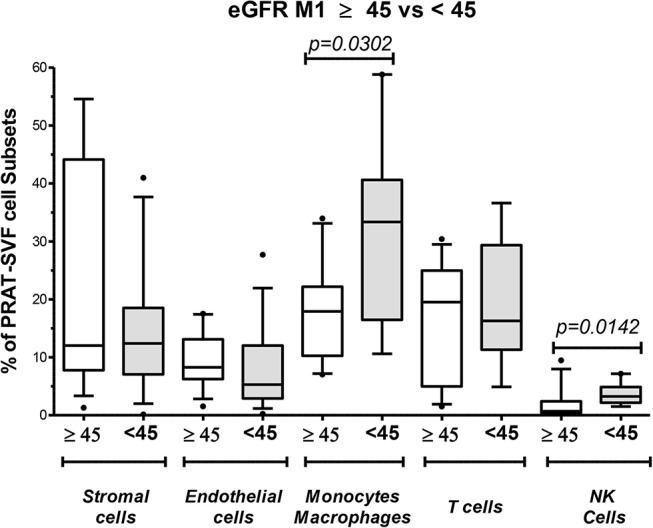
Analysis of perirenal adipose tissue stromal vascular fraction (PRAT-SVF) with lower recovery of graft persisting at M1 post-transplant. Donors were split into two groups according to graft recovery persisting at M1 post-transplant: lower recovery (eGFR M1 < 45) and normal recovery (eGFR > 45). The proportion of cell subsets was analyzed according to this splitting. The eGFR M1 < 45 presented a significantly higher percentage of monocyte/macrophage subset and NK cells. Other cells were similarly distributed between the two groups.

We then investigated whether parameters evaluated in PRAT-SVF could relate to allograft dysfunction during the first month following transplantation. Creatinine levels on D7 and eGFR at M1 post-transplant were significantly correlated with donor age, but did not correlate with cold ischemia time in the studied cohort (Table 3). Early graft dysfunction, as defined by creatinine levels at day 7 and values of eGFR <45 mL/min at one-month (M1) after transplantation, were also correlated with the proportion of PRAT-SVF NK inflammatory cells and monocytes/macrophages (Table 3 and Figure 6) in univariate analysis. We used ROC curve analysis to set a 1.5% NK cell threshold in PRAT-SVF associated with lower CKD at M1 (area under ROC curve = 0.82, sensitivity 100%, specificity 75%). Interestingly, logistic regression models further showed that a percentage of NK cells > to this 1.5 threshold value of NK cells observed in pre-transplant donor PRAT-SVF, was an independent factor associated with lowered graft function recovery (eGFR <45 or 60 at 1-month post-transplant), regardless of the HTA status of the donor (Table 4).

**Table 3:** Analysis of parameters correlating with early graft function at Day 7 (D7) and 1 month (M1) post-transplant.

	**Serum creatinine Day 7**		**eGFR M1**	
**PRAT-SVF donor**	**Spearman r**	***P*-value**	**Spearman r**	***P*-value**
**DONOR CHARACTERISTICS**				
Donor Age	0.3725	0.006	−0.5371	0.0001
Cold Ischemia time (hours)	0.2666	0.0802	ns	ns
**PRAT-SVF IMMUNE CELLS INFILTRATION**				
% CD45+CD14+ Monocytes/macrophages	0.4461	0.0289	−0.4874	0.0214
% CD3-CD56+ NK cells	0.3991	0.0533	−0.4485	0.0363
% CD3+ T cells	0.34	0.104	ns	ns

*PRAT, PeriRenal Adipose Tissue; SVF, Stromal Vascular Fraction; GFR, Glomerular Filtration Rate; Ns, Non-significant*.

**Table 4:** Logistic regression models linking the presence of PRAT-SVF NK cell infiltrates to lowered allograft function evaluated 1-month (M1) post-transplantation.

	**Odds Ratio**	**Std. Err**.	**z**	***p***	**[95% Conf. Interval]**
**Predictive factors of CKD <45 mL/min M1**					
NK SVF ≥1.5%	25.6	33.5	2.48	0.01	[1.9–332.4]
Donor HTA	6.7	10.1	1.26	0.21	[0.3–130.2]
**Predictive factors of CKDM1 <60 mL/min M1**					
NK SVF ≥ 1.5%	10.6	11.2	2.2	0.03	[1.3–84.8]
Donor HTA	4.4	5.9	1.1	0.27	[0.3–61.7]

## Discussion

Taking advantage of the accessibility of donor perirenal adipose tissue, our study is the first to evidence significant changes in the cellular and molecular features that characterize the PRAT-SVF of marginal donors. Compared with optimal donors, marginal donors exhibited significant alterations in the cellular composition of PRAT-SVF that notably comprised an increase in immune-cell infiltrates and levels of transcripts encoding inflammatory chemokines/cytokines. This molecular inflammatory signature was impacted by donor age and could be further associated with early graft dysfunction.

The cell subset distribution in PRAT-SVF of ECD transplants indicated an imbalance between pro and anti-inflammatory cells. ECD donor PRAT-SVF showed a lowered proportion of stromal/mesenchymal cells, recently identified as an immunomodulatory cell compartment of the perirenal adipose tissue (34), and a higher proportion of immune NK lymphocyte infiltrates. Interestingly, enhanced representation of NK and T cells in PRAT-SVF was identified as an age-related specific feature. These results are in line with experimental studies suggesting that kidneys from older donors are more immunogenic and induce increased T-cell responses than kidneys from young donors (24).

Consistently, major changes in the transcriptomic signature of ECD donors were found to be related to upregulation of inflammatory pathways. Among the most up-regulated genes was CXCL1, which is also known as GROα. CXCL1 is a pro-inflammatory chemokine that binds to CXCR2 to promote neutrophil chemotaxis. CXCL1-dependent neutrophil accumulation in a kidney transplant after reperfusion is an important predictor of delayed graft function (35, 36)_._ CXCL1 has also been associated with various inflammatory kidney diseases such acute kidney ischemia and glomerulonephritis (37) and progression of chronic kidney disease (38). Inhibition of CXCR2 prevents kidney graft function deterioration owing to ischemia/reperfusion (39). Our transcriptional analysis also demonstrated that upregulation of the CCL4 chemokine and IL1-beta were associated with the ECD profile. This upregulation was consistent with an enhanced proportion of NK cells infiltrating ECD donor PRAT-SVF and previous data reporting the activation of a CCL4 and IFN-γ dependent pathway in patients with kidney graft rejection (40). Although extrapolation of these observations in donor PRAT-SVF could not be matched with those occurring in the pre-transplant biopsy, these results corroborate previous findings that identify the NKG2D activating receptor as a candidate marker of kidney graft quality in pre-transplant biopsy specimens from donors over 55 years (41).

In kidney transplant recipients, innate NK cells have recently been identified as a key effector mechanism regulating the level of endothelial lesion and repair as well as vascular rejection and allograft vasculopathy (11, 25, 42–44). NK cells have also been reported to contribute to immune senescence in kidney transplant candidates (45). Our observations suggest that PRAT-SVF recruitment of donor NK cells could also promote pro-inflammatory signals that affect the vascular homeostasis of a marginal transplant prior transplantation. While the quantitative distribution of PRAT-SVF endothelial cells was comparable in ECD and non-ECD donors, we observed a high inter-individual variability in PRAT-SVF angiogenic activity, that did not reach significance between the ECD and non-ECD groups analyzed in the present study. Such heterogeneity among donors has already been reported for mesenchymal stem cells (26). However, in line with this result, the study by Aird et al. did not evidence major changes in the angiogenic potential of AT-SVF with aging except a delayed phase of neovessels maturation *in vivo* (46). However, we observed that the percentage of NK cells in PRAT-SVF was inversely correlated with the angiogenic potential, suggesting that, at an individual level, donor-dependent NK cell activation could also provide an inflammatory environment that favors endothelial vulnerability prior to transplantation.

Importantly, among the parameters analyzed with PRAT-SVF, the proportion of NK cells was identified as associated to graft dysfunction evaluated at 7 days and 1-month post transplantation, indicating a potential impact on the clinical outcome of marginal transplants from aging donors. These findings make it possible to speculate that the heterogeneity of inflammatory cytokine overexpression and age-dependent NK cell activation in ECD transplants could contribute to shaping allograft immunogenicity by perpetuating immune cell recruitment and activation, thereby rendering the endothelial cells of the graft more vulnerable to further exposure to ischemia/reperfusion, uremic, and alloimmune inflammatory stresses. These markers could thus be regarded as potential molecular targets for strategies enabling to reduce inflammation in ECD transplants.

Our study presents limitations since the unavailability of pre-transplant renal biopsies did not enable evaluation of the specific features that characterized PRAT-SVF in marginal donors which could be extended to the renal parenchyma (47). However, these data provided evidence that tissue recruitment of donor NK cells may *per se* participate in the pre-conditioning of transplant vulnerability and quality and prompt further investigation of the clinical relevance of such biomarkers in larger cohorts. This work could introduce PRAT-SVF as an innovative and less invasive approach with added value in terms of feasibility compared with pre-implantation biopsies and also document the specific features that characterize perirenal fat (47, 48). Another limitation is that the RNAseq and the functional analysis of the angiogenic capacity of donor-derived cells were performed on the whole PRAT-SVF and not on individual SVF sorted cell types. This experimental design allowed to integrate the cellular crosstalk between SVF cell subsets and prevented a potential bias associated with cell subset isolation and expansion *in vitro*. However, it did not allow to define if the observed changes resulted from alteration of PRAT-SVF composition or from a specific imprint of the ECD microenvironment on a given cell type. These data call for a more in-depth analysis using a single cell approach characterizing the transcriptomic profile of PRAT-SVF specific to the microenvironment of the ECD donor and the specific study of mesenchymal and endothelial cells purified from perirenal SVF. Future single cell analysis approaches and comparative analyses of purified endothelial and mesenchymal cells isolated from PRAT-SVF ECD and optimal donors would be of value to provide additional mechanistic clues.

Although the immediate implications of PRAT-SVF are not compatible with the current clinical setting of transplantation, our work may open perspectives to target inflammatory pathways in order to reduce donor-related inflammation before transplantation during the dynamic hypothermic machine perfusion process with the aim to optimize transplant quality.

## Conclusion

Our results argue in favor of a donor-dependent inflammation-driven alteration of pre-transplant allograft quality and identify NK cells as potential effectors of pro-inflammatory remodeling mechanisms that can affect the function of marginal elderly transplants.

## Data Availability Statement

The raw data supporting the conclusions of this article will be made available by the authors, without undue reservation, to any qualified researcher. The RNAseq data has been submitted to the GEO repository under accession numbers GSE140122.

## Ethics Statement

The study was approved by the National Ethics Committee of the Agence de Biomédecine (ABM), the National Ministry of Research and adhered to the Jardé Law on human investigation. All procedures were conducted in compliance with the Declarations of Helsinki and Istanbul. Data were prospectively and anonymously collected in a dedicated database for the exclusive access of the authorized authors.

## Author Contributions

RB, PP, GK, EL, and FS contributed to the conception and design of the study. RB, GK, MM, EL, BG, TL, and SB enrolled subjects into the study, collected primary data. LL, PF, BG, SS, JM, LG, and LA performed the experiments. PP reviewed the statistical analysis and wrote the manuscript. FD and FS wrote sections of the manuscript. All authors contributed to manuscript revision and read and approved the submitted version.

### Conflict of Interest

The authors declare that the research was conducted in the absence of any commercial or financial relationships that could be construed as a potential conflict of interest.

## Abbreviations

ABM: Agence de la Biomédecine
DGF: delayed graft function
ECD: expanded criteria donors
ECFC: endothelial colony forming cells
GFR: glomerular filtration rate
LD: living donor
NK: Natural Killer cells
Non-ECD: non-expanded criteria donors
PRAT: perirenal adipose tissue
SGF: slow graft function
SVF: stromal vascular fraction.
