# Supplementary material for: Perirenal Adipose Tissue Displays an Age-Dependent Inflammatory Signature Associated With Early Graft Dysfunction of Marginal Kidney Transplants
Source: Front Immunol. 2020 Mar 17;11:445. doi: 10.3389/fimmu.2020.00445 (PMC7089962; doi:10.3389/fimmu.2020.00445)
Supplement: Supplementary file 1 [file Data_Sheet_1.docx]

Supplementary Material

**Supplementary Figures**

**Supplementary Figure 1: gating strategy for flow cytometry analysis of PRAT-SVF cell subsets**


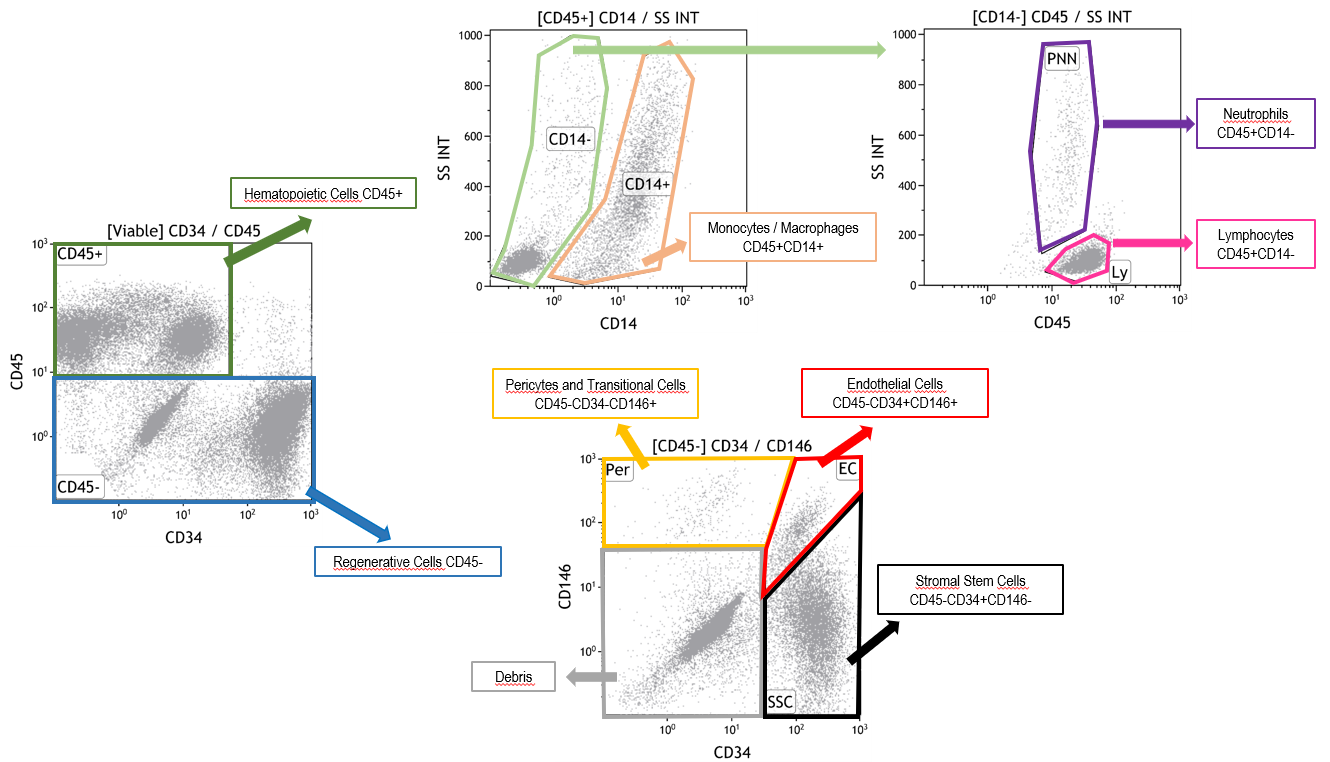


Viable nucleated events were selected as positive DRAQ5+ NucBlue-negative events. Among them, the CD45 marker separated the population of hematopoietic CD45+ cells from the regenerative cells CD45-. Using the antibody mix 1, three regenerative cell subsets were identified as mesenchymal stem/stromal cells (CD45-CD34-CD146), endothelial cells (CD45-CD34-CD146+) and a pool of pericytes and transitional cells (CD45-CD34-CD146+). Using the antibody mix 2, CD14 marker made it possible to discriminate the monocyte/macrophage subset (CD45+CD14+) and the CD45+CD14- population was analyzed based on size scatter to discriminate lymphocytes and neutrophils. In addition, multiparameter analysis allowed further characterization of lymphocyte subsets and identification of T cells (CD45+CD3+) and NK cells (CD45+CD3-CD56+).

**Supplementary Figure 2: In vitro angiogenic assay of collagen gel-embedded spheroids** **of PRAT-SVF from 2 representative ECD vs. non-ECD donors.**

Example images showing spheroids with vascular sprouts stained for F-actin using phalloidin (green) and nuclei using DAPI (blue) (original magnification x20; scale bars, 100µm).

## Supplementary Tables

**Supplementary Table 1: Monoclonal antibodies used for phenotypic characterization of PRAT-SVF cell subsets.**

| **Mix** | **Specificity** | **Supplier** | **Reference** | **Volume** | **Concentration of conjugate (µg/mL)** |
| --- | --- | --- | --- | --- | --- |
| 1 | CD146-PE | Beckman Coulter | PN A07483 | 10μL | 6.25 |
|  | CD34-ECD | Beckman Coulter | IM2709U | 10μL | 15.0 |
|  | CD45-PC5 | Beckman Coulter | A07785 | 10μL | 50.0 |
|  | DRAQ5 | eBioscience | 65-0880-96 | 10μL | 82.5 |
| 2 | CD14-FITC | Beckman Coulter | B36297 | 10μL | 12.5 |
|  | CD34-ECD | Beckman Coulter | IM2709U | 10μL | 15.0 |
|  | CD45-PC5 | Beckman Coulter | A07785 | 10μL | 50.0 |
|  | CD56-PC7 | Beckman Coulter | A21692 | 5μL | 12.5 |
|  | CD3APC-Alexa Fluor 750 | Beckman Coulter | A94680 | 5μL | 100.0 |
|  | DRAQ5 | eBioscience | 65-0880-96 | 10μL | 82.5 |

**Supplementary Table 2: Genes selected for qPCR validation in PRAT SVF.**

| *CXCL1* | Forward | AAGCTTGCCTCAATCCTGCA |
| --- | --- | --- |
| *CXCL1* | Reverse | GGCCTCTGCAGCTGTGTCTC |
| *VWA7* | Forward | TCCTCACCTCTGGCTACTTTGG |
| *VWA7* | Reverse | GGGATGTGCTGTCCTTGTTGAT |
| *CCL4* | Forward | GAAGCTCTGCGTGACTGTCCT |
| *CCL4* | Reverse | ACAGCTGGCTGGGAGCAGA |
| *IL1-β* | Forward | GAAGAAAGTAATGACAAAATACCTGTGGCC |
| *IL1-β* | Reverse | CCACATTCAGCACAGGACTCTCTGG |
| *IFN−γ* | Forward | TGCAGAGCCAAATTGTCTCCTT |
| *IFN-γ* | Reverse | CATGTATTGCTTTGCGTTGGAC |
| *NKG2D* | Forward | GGTATGAGAGCCAGGCTTCTTGT |
| *NKG2D* | Reverse | CTTTACACAGTCCTTTGCATGCA |
| *FGFR2* | Forward | AATCACACGTACCACCTGGATG |
| *FGFR2* | Reverse | ACTCTACGTCTCCTCCGACCAC |

**Supplementary Table 3: Characteristics of PRAT-SVF samples (n=10) of 5 ECD and 5 non ECD donors analyzed by RNA-Seq transcriptomic approaches**

| **Sample ID** | **ECD/ non ECD** | **Donor Age** | **Donor HTA** |
| --- | --- | --- | --- |
| PRAT-SVF S01 | ECD | 73 | 1 |
| PRAT-SVF S02 | ECD | 88 | 1 |
| PRAT-SVF S03 | ECD | 53 | 0 |
| PRAT-SVF S04 | ECD | 63 | 1 |
| PRAT-SVF S05 | ECD | 78 | 1 |
| PRAT-SVF S06 | non ECD | 17 | 0 |
| PRAT-SVF S07 | non ECD | 44 | 0 |
| PRAT-SVF S08 | non ECD | 48 | 0 |
| PRAT-SVF S09 | non ECD | 16 | 0 |
| PRAT-SVF S10 | non ECD | 48 | 1 |

**Supplementary Table 4: Up-regulated genes in PRAT-SVF of ECD patients.**

| ensembl_gene_id | symbol | logFC | P-Value |
| --- | --- | --- | --- |
| ENSG00000109424 | UCP1 | 4.92151268 | 0.00476949 |
| ENSG00000159251 | ACTC1 | 4.03735639 | 0.00416316 |
| ENSG00000117154 | IGSF21 | 3.37728654 | 0.00051096 |
| ENSG00000164825 | DEFB1 | 3.34937391 | 0.00375952 |
| ENSG00000175426 | PCSK1 | 3.2771247 | 0.00192749 |
| ENSG00000157766 | ACAN | 3.23866513 | 0.01218911 |
| ENSG00000134339 | SAA2 | 3.06451677 | 0.0204919 |
| ENSG00000248923 | MTND5P11 | 2.9062242 | 0.0038216 |
| ENSG00000170893 | TRH | 2.8280618 | 0.00496247 |
| ENSG00000231475 | IGHV4-31 | 2.74025036 | 0.00490906 |
| ENSG00000163958 | ZDHHC19 | 2.67416455 | 0.00734053 |
| ENSG00000186191 | BPIFB4 | 2.61224556 | 0.01165984 |
| ENSG00000221957 | KIR2DS4 | 2.59081872 | 0.01371028 |
| ENSG00000244921 | MTCYBP18 | 2.54317408 | 0.03024677 |
| ENSG00000189068 | VSTM1 | 2.4870869 | 0.01080293 |
| ENSG00000211972 | IGHV3-66 | 2.40198072 | 0.04965893 |
| ENSG00000211640 | IGLV6-57 | 2.36527045 | 0.01800361 |
| ENSG00000125337 | KIF25 | 2.35522838 | 0.01649053 |
| ENSG00000170439 | METTL7B | 2.29885376 | 0.02018673 |
| ENSG00000211662 | IGLV3-21 | 2.28598779 | 0.02662165 |
| ENSG00000103522 | IL21R | 2.28150199 | 0.01226093 |
| **ENSG00000125538** | **IL1B** | **2.27798096** | **0.00446864** |
| ENSG00000124102 | PI3 | 2.27487573 | 0.02659747 |
| ENSG00000108342 | CSF3 | 2.25624006 | 0.01930433 |
| ENSG00000138207 | RBP4 | 2.21434005 | 0.00489522 |
| ENSG00000211892 | IGHG4 | 2.20158264 | 0.01945059 |
| ENSG00000173391 | OLR1 | 2.18755883 | 0.00332571 |
| ENSG00000211685 | IGLC7 | 2.18505624 | 0.02788426 |
| ENSG00000113578 | FGF1 | 2.17519186 | 8.0142E-05 |
| ENSG00000211651 | IGLV1-44 | 2.16230925 | 0.00584912 |
| **ENSG00000111537** | **IFNG** | **2.15258735** | **0.01388106** |
| ENSG00000137869 | CYP19A1 | 2.14050866 | 0.02860994 |
| ENSG00000235576 | LINC01871 | 2.13752474 | 0.00446469 |
| ENSG00000104918 | RETN | 2.10436452 | 0.00361614 |
| ENSG00000276085 | CCL3L3 | 2.10322323 | 0.00489105 |
| ENSG00000084674 | APOB | 2.09877576 | 0.02924821 |
| ENSG00000262902 | MTCO1P40 | 2.06198835 | 0.03100354 |
| ENSG00000250771 | AC106865.1 | 2.04892397 | 0.02063052 |
| ENSG00000101443 | WFDC2 | 2.03764325 | 0.00438357 |
| ENSG00000258227 | CLEC5A | 2.02725457 | 0.0058097 |
| ENSG00000224114 | AL591846.1 | 2.01934572 | 0.03254768 |
| ENSG00000276758 | AC245884.12 | 2.01517265 | 0.00219777 |
| ENSG00000170858 | LILRP2 | 2.01279833 | 0.00243237 |
| ENSG00000176083 | ZNF683 | 2.01029136 | 0.03507223 |
| ENSG00000184106 | TREML3P | 1.98135342 | 0.03667612 |
| ENSG00000143184 | XCL1 | 1.97009314 | 0.03501285 |
| ENSG00000180739 | S1PR5 | 1.96856053 | 0.04576294 |
| ENSG00000276070 | CCL4L2 | 1.9637639 | 0.00195067 |
| ENSG00000178803 | ADORA2A-AS1 | 1.96354226 | 0.00323519 |
| ENSG00000117115 | PADI2 | 1.94373255 | 0.00870355 |
| ENSG00000175084 | DES | 1.93537968 | 0.01168516 |
| **ENSG00000163739** | **CXCL1** | **1.91194326** | **1.5103E-05** |
| ENSG00000271503 | CCL5 | 1.91138745 | 0.01818478 |
| ENSG00000139572 | GPR84 | 1.90561189 | 0.00467943 |
| ENSG00000236438 | FAM157A | 1.90371014 | 0.00947386 |
| ENSG00000117560 | FASLG | 1.89770483 | 0.02164447 |
| ENSG00000268734 | AC245128.3 | 1.89629859 | 0.00917452 |
| ENSG00000172724 | CCL19 | 1.89412018 | 0.00170363 |
| **ENSG00000275302** | **CCL4** | **1.86840717** | **0.00183256** |
| ENSG00000267607 | AC011511.5 | 1.868224 | 0.01010632 |
| ENSG00000130487 | KLHDC7B | 1.86790782 | 0.010693 |
| ENSG00000181036 | FCRL6 | 1.86466087 | 0.02998503 |
| ENSG00000273237 | AC004520.1 | 1.85762062 | 0.00299018 |
| ENSG00000162676 | GFI1 | 1.85667547 | 0.0268742 |
| ENSG00000186074 | CD300LF | 1.8513693 | 0.0074912 |
| ENSG00000229331 | GK-IT1 | 1.84176128 | 0.02545407 |
| ENSG00000186431 | FCAR | 1.83915064 | 0.01577757 |
| ENSG00000234389 | AC007278.1 | 1.83039907 | 0.0254064 |
| ENSG00000134042 | MRO | 1.79715037 | 0.00624077 |
| ENSG00000270607 | AC009549.1 | 1.78711331 | 0.0482545 |
| ENSG00000077984 | CST7 | 1.77275631 | 0.01864657 |
| ENSG00000143502 | SUSD4 | 1.76058644 | 0.01862184 |
| **ENSG00000204396** | **VWA7** | **1.75470229** | **0.00031288** |
| ENSG00000134070 | IRAK2 | 1.74999793 | 0.00501854 |
| ENSG00000163534 | FCRL1 | 1.74178559 | 0.04844133 |
| ENSG00000163734 | CXCL3 | 1.74106727 | 0.00246157 |
| ENSG00000184669 | OR7E14P | 1.73875139 | 0.04411161 |
| ENSG00000210184 | MT-TS2 | 1.73266473 | 0.02007769 |
| ENSG00000054938 | CHRDL2 | 1.72574768 | 0.00916207 |
| ENSG00000142549 | IGLON5 | 1.71630336 | 0.03282636 |
| ENSG00000253522 | MIR3142HG | 1.71390719 | 0.01977609 |
| ENSG00000115009 | CCL20 | 1.70493209 | 0.01203622 |
| ENSG00000273338 | AC103591.3 | 1.69755454 | 0.00738671 |
| ENSG00000064886 | CHI3L2 | 1.69420852 | 0.00978334 |
| ENSG00000125735 | TNFSF14 | 1.68122953 | 0.00174145 |
| ENSG00000210154 | MT-TD | 1.67613432 | 0.02637723 |
| ENSG00000186197 | EDARADD | 1.66929192 | 0.02853129 |
| ENSG00000276980 | AC008760.2 | 1.66597877 | 0.0381103 |
| ENSG00000210174 | MT-TR | 1.66298828 | 0.0227418 |
| ENSG00000251230 | MIR3945HG | 1.6559475 | 0.0019555 |
| ENSG00000187116 | LILRA5 | 1.64866642 | 0.00759293 |
| ENSG00000167077 | MEI1 | 1.64692725 | 0.00994904 |
| ENSG00000100450 | GZMH | 1.63368819 | 0.04106828 |
| ENSG00000166920 | C15orf48 | 1.62660158 | 0.0399024 |
| ENSG00000115339 | GALNT3 | 1.60403672 | 0.00043393 |
| ENSG00000224397 | SMIM25 | 1.59536934 | 0.00726345 |
| ENSG00000142512 | SIGLEC10 | 1.58587522 | 0.00336595 |
| ENSG00000233093 | LINC00892 | 1.58363968 | 0.04914678 |
| ENSG00000188404 | SELL | 1.57065788 | 0.04828771 |
| ENSG00000197249 | SERPINA1 | 1.57031718 | 0.00282829 |
| ENSG00000279766 | AC067931.1 | 1.56527235 | 0.03239811 |
| ENSG00000171101 | SIGLEC17P | 1.56151893 | 0.02919633 |
| ENSG00000167604 | NFKBID | 1.54833783 | 0.00865625 |
| ENSG00000122641 | INHBA | 1.5414971 | 0.04747827 |
| ENSG00000184156 | KCNQ3 | 1.53926814 | 0.0163775 |
| ENSG00000210164 | MT-TG | 1.52889036 | 0.04477221 |
| ENSG00000159618 | ADGRG5 | 1.52307684 | 0.04733401 |
| ENSG00000105374 | NKG7 | 1.52031768 | 0.03160633 |
| ENSG00000154099 | DNAAF1 | 1.51605125 | 0.0151477 |
| ENSG00000145287 | PLAC8 | 1.51509177 | 0.00585256 |
| ENSG00000053438 | NNAT | 1.50624708 | 0.04309935 |

**Supplementary Table 5: transcriptomic analysis of ECD PRAT-SVF downregulated genes.**

| ensembl_gene_id | symbol | logFC | P-Value |
| --- | --- | --- | --- |
| ENSG00000187398 | LUZP2 | -5.36424914 | 0.00076617 |
| ENSG00000114771 | AADAC | -4.9424271 | 0.0014306 |
| ENSG00000231852 | CYP21A2 | -4.76314659 | 0.04004016 |
| ENSG00000076864 | RAP1GAP | -4.68323179 | 0.00151214 |
| ENSG00000165449 | SLC16A9 | -4.44972296 | 0.0039528 |
| ENSG00000187957 | DNER | -4.37728702 | 0.00536245 |
| ENSG00000188488 | SERPINA5 | -4.20382768 | 0.009073 |
| ENSG00000171303 | KCNK3 | -4.17192107 | 0.0186307 |
| ENSG00000170558 | CDH2 | -4.14958454 | 0.03183127 |
| ENSG00000066468 | **FGFR2** | -4.06362665 | 0.00308756 |
| ENSG00000178947 | SMIM10L2A | -4.06049774 | 0.00513028 |
| ENSG00000082482 | KCNK2 | -4.0009516 | 0.01258568 |
| ENSG00000140459 | CYP11A1 | -3.97005629 | 0.01571426 |
| ENSG00000104833 | TUBB4A | -3.96334949 | 0.02384085 |
| ENSG00000136546 | SCN7A | -3.95493089 | 0.00017238 |
| ENSG00000242265 | PEG10 | -3.83226472 | 0.00217681 |
| ENSG00000178821 | TMEM52 | -3.77906858 | 0.00367806 |
| ENSG00000101311 | FERMT1 | -3.65877305 | 0.0079296 |
| ENSG00000130988 | RGN | -3.6460339 | 0.00566248 |
| ENSG00000136999 | NOV | -3.61962151 | 0.02125477 |
| ENSG00000123572 | NRK | -3.56824205 | 0.01163051 |
| ENSG00000142494 | SLC47A1 | -3.56769112 | 0.00245917 |
| ENSG00000091129 | NRCAM | -3.56729638 | 0.03312028 |
| ENSG00000103034 | NDRG4 | -3.55604154 | 0.00305055 |
| ENSG00000171724 | VAT1L | -3.47311759 | 0.02961122 |
| ENSG00000152092 | ASTN1 | -3.43775454 | 0.01160592 |
| ENSG00000161513 | FDXR | -3.27851967 | 0.00927092 |
| ENSG00000023171 | GRAMD1B | -3.22641565 | 0.00641444 |
| ENSG00000169297 | NR0B1 | -3.21093983 | 0.01422484 |
| ENSG00000085563 | ABCB1 | -3.13385146 | 0.02034561 |
| ENSG00000114279 | FGF12 | -3.12864211 | 0.01648265 |
| ENSG00000151572 | ANO4 | -3.07559662 | 0.00261685 |
| ENSG00000055732 | MCOLN3 | -2.98765892 | 0.00242962 |
| ENSG00000171798 | KNDC1 | -2.95930881 | 0.02151132 |
| ENSG00000016402 | IL20RA | -2.92363984 | 0.00348016 |
| ENSG00000169330 | KIAA1024 | -2.91405274 | 0.01321805 |
| ENSG00000108176 | DNAJC12 | -2.89402628 | 0.01280445 |
| ENSG00000106236 | NPTX2 | -2.87412235 | 0.01582357 |
| ENSG00000005471 | ABCB4 | -2.82664981 | 0.00584697 |
| ENSG00000162494 | LRRC38 | -2.81561567 | 0.02201354 |
| ENSG00000016391 | CHDH | -2.72939975 | 0.00329425 |
| ENSG00000139344 | AMDHD1 | -2.7233537 | 0.00274244 |
| ENSG00000145284 | SCD5 | -2.70188699 | 0.00573282 |
| ENSG00000168280 | KIF5C | -2.69923416 | 0.00577844 |
| ENSG00000137714 | FDX1 | -2.68083006 | 0.00889928 |
| ENSG00000149809 | TM7SF2 | -2.66839053 | 0.022587 |
| ENSG00000153002 | CPB1 | -2.66156227 | 0.02292372 |
| ENSG00000165995 | CACNB2 | -2.60635767 | 0.04215117 |
| ENSG00000107518 | ATRNL1 | -2.49328072 | 0.01886324 |
| ENSG00000197415 | VEPH1 | -2.47599946 | 0.03266384 |
| ENSG00000170500 | LONRF2 | -2.47246957 | 0.0091686 |
| ENSG00000166473 | PKD1L2 | -2.46610196 | 0.01272944 |
| ENSG00000100346 | CACNA1I | -2.46387457 | 0.01691785 |
| ENSG00000139044 | B4GALNT3 | -2.45678723 | 0.019544 |
| ENSG00000198300 | PEG3 | -2.44961216 | 0.02106016 |
| ENSG00000116133 | DHCR24 | -2.39790983 | 0.02530133 |
| ENSG00000182013 | PNMA8A | -2.38354435 | 0.02303624 |
| ENSG00000196482 | ESRRG | -2.38195953 | 0.032999 |
| ENSG00000147180 | ZNF711 | -2.3554144 | 0.0025019 |
| ENSG00000196517 | SLC6A9 | -2.34831002 | 0.00872078 |
| ENSG00000168824 | NSG1 | -2.33677586 | 0.03722948 |
| ENSG00000230615 | AL139220.2 | -2.30902372 | 0.01712274 |
| ENSG00000123892 | RAB38 | -2.28115835 | 0.00854052 |
| ENSG00000198753 | PLXNB3 | -2.28107457 | 0.01338049 |
| ENSG00000206432 | TMEM200C | -2.25651308 | 0.00792461 |
| ENSG00000137959 | IFI44L | -2.2396319 | 0.0076925 |
| ENSG00000176909 | MAMSTR | -2.21600395 | 0.02111418 |
| ENSG00000149294 | NCAM1 | -2.20923601 | 0.01046559 |
| ENSG00000137819 | PAQR5 | -2.19777383 | 0.00828275 |
| ENSG00000164542 | KIAA0895 | -2.17478247 | 0.02985308 |
| ENSG00000109072 | VTN | -2.17063967 | 0.0394721 |
| ENSG00000171551 | ECEL1 | -2.16168802 | 0.04582218 |
| ENSG00000135111 | TBX3 | -2.15923921 | 0.02133804 |
| ENSG00000060718 | COL11A1 | -2.14396939 | 0.01580144 |
| ENSG00000282608 | ADORA3 | -2.13568179 | 0.003555 |
| ENSG00000164171 | ITGA2 | -2.11635851 | 0.02908388 |
| ENSG00000188707 | ZBED6CL | -2.10460253 | 0.016058 |
| ENSG00000134321 | RSAD2 | -2.09293174 | 0.01519277 |
| ENSG00000105767 | CADM4 | -2.08215305 | 0.01520315 |
| ENSG00000160867 | FGFR4 | -2.07017355 | 0.00549197 |
| ENSG00000091428 | RAPGEF4 | -2.03122755 | 0.02597408 |
| ENSG00000088538 | DOCK3 | -2.02685601 | 0.02404185 |
| ENSG00000103184 | SEC14L5 | -2.02385925 | 0.0131368 |
| ENSG00000177875 | CCDC184 | -1.9812525 | 0.04229052 |
| ENSG00000138795 | LEF1 | -1.96588392 | 0.01263177 |
| ENSG00000247095 | MIR210HG | -1.93952218 | 0.01367343 |
| ENSG00000181754 | AMIGO1 | -1.93747189 | 0.01077454 |
| ENSG00000185745 | IFIT1 | -1.92331869 | 0.01420219 |
| ENSG00000096696 | DSP | -1.893135 | 0.02568657 |
| ENSG00000073060 | SCARB1 | -1.87045279 | 0.04396673 |
| ENSG00000184838 | PRR16 | -1.86954442 | 0.022482 |
| ENSG00000118507 | AKAP7 | -1.8631107 | 0.01677962 |
| ENSG00000198417 | MT1F | -1.85520989 | 0.01271247 |
| ENSG00000241404 | EGFL8 | -1.84758582 | 0.00064546 |
| ENSG00000183287 | CCBE1 | -1.83382524 | 0.02873862 |
| ENSG00000135519 | KCNH3 | -1.83056638 | 0.02904505 |
| ENSG00000146151 | HMGCLL1 | -1.81783164 | 0.03293177 |
| ENSG00000154654 | NCAM2 | -1.81327439 | 0.01415686 |
| ENSG00000148082 | SHC3 | -1.79944208 | 0.03906124 |
| ENSG00000196090 | PTPRT | -1.79115329 | 0.02402944 |
| ENSG00000170899 | GSTA4 | -1.76894849 | 0.04041151 |
| ENSG00000100285 | NEFH | -1.75664088 | 0.00524803 |
| ENSG00000108924 | HLF | -1.75452714 | 0.04727939 |
| ENSG00000054690 | PLEKHH1 | -1.75346187 | 0.03627113 |
| ENSG00000174938 | SEZ6L2 | -1.74934979 | 0.03695324 |
| ENSG00000161249 | DMKN | -1.7440908 | 0.02942101 |
| ENSG00000144893 | MED12L | -1.73419557 | 0.00710135 |
| ENSG00000280067 | AC023818.2 | -1.71940941 | 0.00691453 |
| ENSG00000162522 | KIAA1522 | -1.71913855 | 0.01783414 |
| ENSG00000162415 | ZSWIM5 | -1.70790141 | 0.02586425 |
| ENSG00000138642 | HERC6 | -1.69891147 | 0.02221943 |
| ENSG00000063587 | ZNF275 | -1.67978049 | 0.01129971 |
| ENSG00000062524 | LTK | -1.66429453 | 0.01135681 |
| ENSG00000177570 | SAMD12 | -1.62585271 | 0.02524883 |
| ENSG00000167178 | ISLR2 | -1.62162652 | 0.00184231 |
| ENSG00000203722 | RAET1G | -1.60728467 | 0.03932732 |
| ENSG00000005187 | ACSM3 | -1.589965 | 0.01595351 |
| ENSG00000140009 | ESR2 | -1.58710488 | 0.03258848 |
| ENSG00000185909 | KLHDC8B | -1.58380972 | 0.0330691 |
| ENSG00000085662 | AKR1B1 | -1.58347687 | 0.02666033 |
| ENSG00000157617 | C2CD2 | -1.57779728 | 0.02322655 |
| ENSG00000253636 | AC022893.1 | -1.56797085 | 0.02195798 |
| ENSG00000134326 | CMPK2 | -1.56567194 | 0.00832738 |
| ENSG00000138829 | FBN2 | -1.56068228 | 0.01460497 |
| ENSG00000091622 | PITPNM3 | -1.55082939 | 0.04663359 |
| ENSG00000165912 | PACSIN3 | -1.54534874 | 0.02980047 |
| ENSG00000184792 | OSBP2 | -1.54426652 | 0.01563336 |
| ENSG00000118898 | PPL | -1.53648715 | 0.03842144 |
| ENSG00000008277 | ADAM22 | -1.52946197 | 0.01625333 |
| ENSG00000006606 | CCL26 | -1.52902899 | 0.03986096 |
| ENSG00000186998 | EMID1 | -1.51930276 | 0.04663994 |
| ENSG00000143674 | MAP3K21 | -1.51307433 | 0.01604418 |
| ENSG00000160345 | C9orf116 | -1.50255845 | 0.04701041 |
| ENSG00000206190 | ATP10A | -1.50181772 | 0.02641282 |

**Supplementary Table 6: Gene Ontology analysis for classification of the genes upregulated in ECD based on the biological process.**

**Supplementary Table 7: Gene Ontology analysis for classification of the genes downregulated in ECD based on the biological process.**

**Supplementary Table 8: Pathways enriched in genes upregulated in ECD samples by** **KEGG analysis.**

| KEGG | TERM | Count | PValue Enrichment | Genes |
| --- | --- | --- | --- | --- |
| hsa04060 | Cytokine-cytokine receptor interaction | 16 | 1.9687820247852136E-14 | **CXCL1,** CSF3, CXCL3, IL21R, TNFSF14, CCL19, FASLG, CCL4L2, CCL5, CCL4, INHBA, CCL20, CCL3L3, **IFNG, IL1B,** XCL1 |
| hsa04062 | Chemokine signaling pathway | 9 | 1.1380038240874998E-6 | CXCL1, CCL20, CXCL3, CCL3L3, CCL19, CCL4L2, CCL5, XCL1, CCL4 |
| hsa04064 | NF-kappa B signaling pathway | 5 | 5.78704922051907E-4 | CCL19, **IL1B**, TNFSF14, CCL4L2, **CCL4** |
| hsa04620 | Toll-like receptor signaling pathway | 5 | 0.0012166942395064819 | CCL3L3, **IL1B,** CCL4L2, CCL5, **CCL4** |
| hsa04668 | TNF signaling pathway | 5 | 0.001259980533966889 | **CXCL1,** CCL20, CXCL3, **IL1B**, CCL5 |
| hsa04623 | Cytosolic DNA-sensing pathway | 4 | 0.0028671328216308776 | **IL1B,** CCL4L2, CCL5, **CCL4** |
| hsa05332 | Graft-versus-host disease | 3 | 0.009514100553652009 | **IFNG, IL1B**, FASLG |
| hsa04621 | NOD-like receptor signaling pathway | 3 | 0.026027375487248084 | CXCL1, **IL1B,** CCL5 |

**Supplementary Table 9: Pathways enriched in genes downregulated in ECD samples by** **KEGG analysis.**

| KEGG | Term | Count | PValue Enrichment | Genes |
| --- | --- | --- | --- | --- |
| bta05412 | Arrhythmogenic right ventricular cardiomyopathy (ARVC) | 5 | 7.327432397693488E-4 | LEF1, ITGA2, CACNB2, DSP, CDH2 |
| bta04925 | Aldosterone synthesis and secretion | 5 | 0.001523722310987306 | CYP11A1, CYP21A2, CACNA1I, SCARB1, KCNK3 |
| bta04015 | Rap1 signaling pathway | 5 | 0.04627705423040515 | FGFR2, FGFR4, RAP1GAP, RAPGEF4, FGF12 |

**Supplementary Table 10: Up-regulated genes selected for qPCR validation in PRAT SVF.**

Relative median values of transcript levels (25-75 interquartile ranges) and P values
